# Supplementary material for: N-linked Fc glycosylation is not required for IgG-B-cell receptor function in a GC-derived B-cell line
Source: Nat Commun. 2024 Jan 9;15:393. doi: 10.1038/s41467-023-44468-5 (PMC10776614; doi:10.1038/s41467-023-44468-5)
Supplement: Supplementary file 1 — Supplementary Information file [file 41467_2023_44468_MOESM1_ESM.pdf]

Supplementary Information for

Title

N-linked Fc glycosylation is not required for IgG-B-cell receptor function in a GC-derived B-cell line

Author list

Theresa Kissel[1, †,*], Veerle F.A.M. Derksen[1, †], Arthur E.H. Bentlage[2], Carolien Koeleman[3], Lise Hafkenscheid[1], Diane van der Woude[1], Manfred Wuhrer[3], Gestur Vidarsson[2], Rene E.M. Toes[1,*]

Affiliations

[1] Department of Rheumatology, Leiden University Medical Center, 2333 ZA Leiden, The Netherlands

[2] Department of Experimental Immunohematology, Sanquin Research and Landsteiner Laboratory, Amsterdam University Medical Center, University of Amsterdam, 1006 AD Amsterdam, The Netherlands

[3] Center for Proteomics and Metabolomics, Leiden University Medical Center, 2333 ZA Leiden, The Netherlands

[†] These authors contributed equally: Theresa Kissel, Veerle F.A.M. Derksen

[*] Correspondence should be addressed to T.K. (email: T.Kissel@lumc.nl) or to R.E.M.T. (email: R.E.M.Toes@lumc.nl)

Supplementary Figures

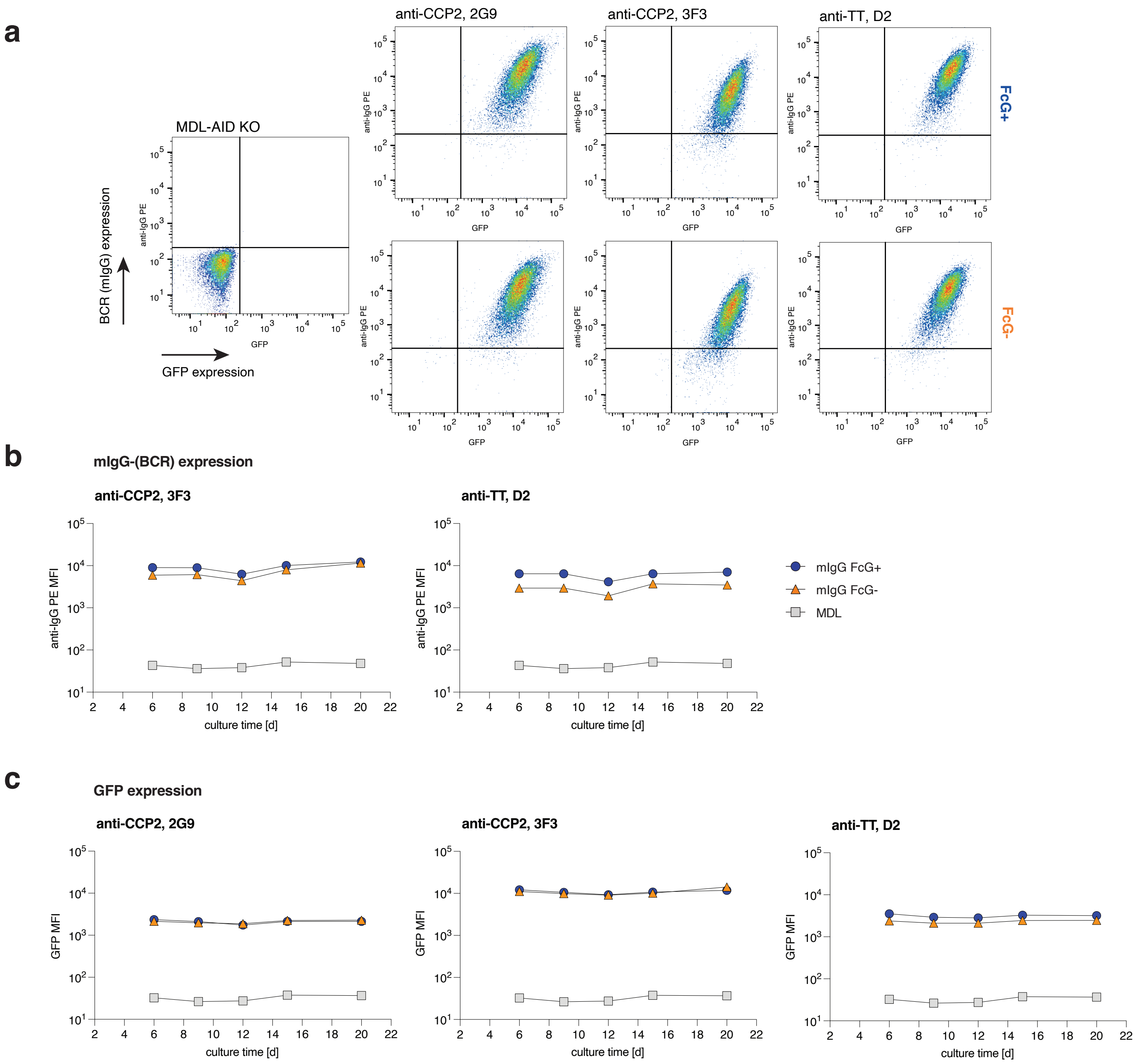

Supplementary Figure 1. BCR and GFP expression of B-cell lines. a Gating strategy to determine GFP and mIgG-BCR expression of transduced FcG+ and FcG- 2G9, 3F3 and D2 Ramos B cell lines. **b** mIgG-(BCR) and **c** GFP expression of anti-CCP2 (3F3) and anti-TT (D2) FcG+ and FcG- and MDL-AID KO B cells over culture time (20 days). Source data are provided as a Source Data file.

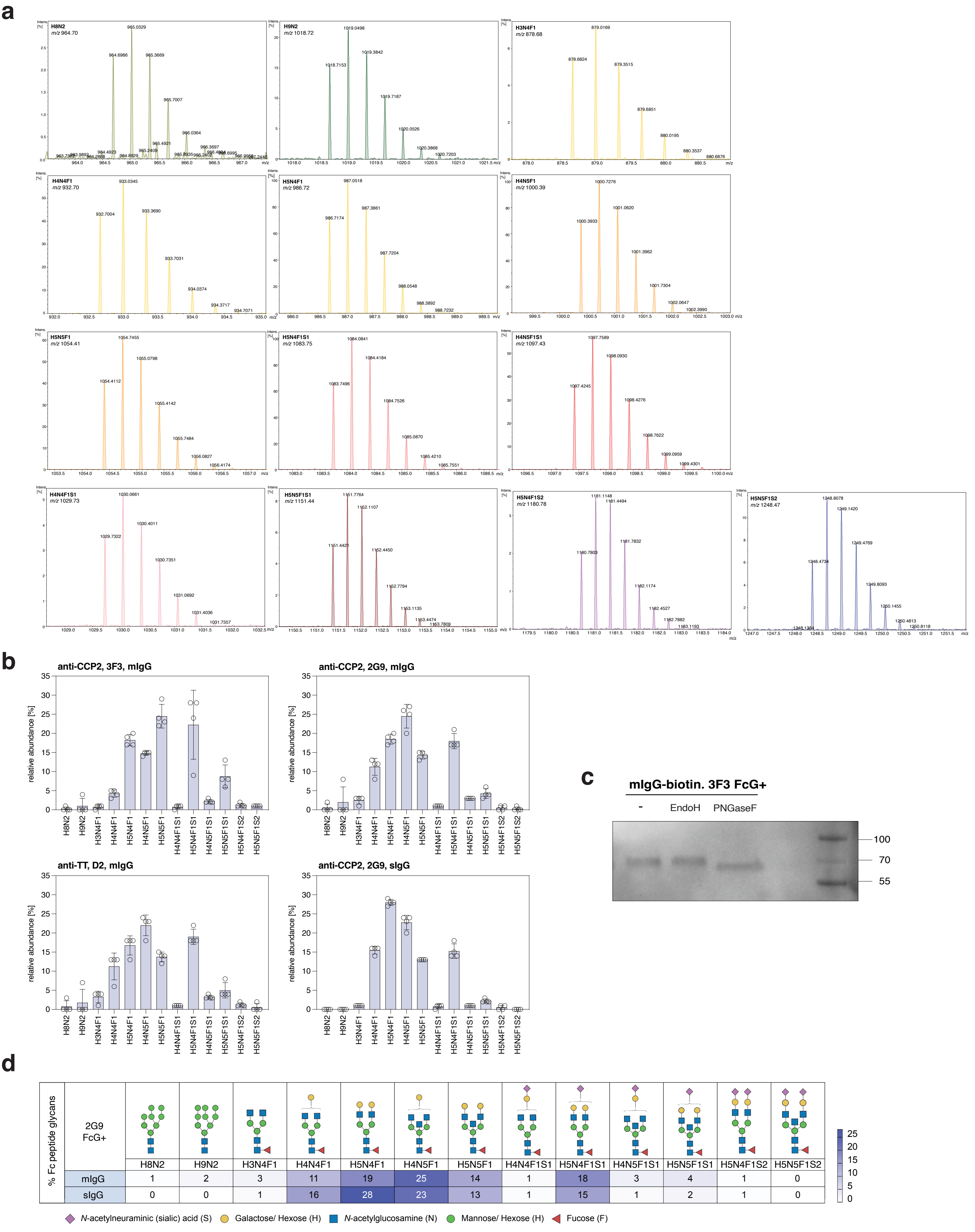

Supplementary Figure 2. Fc peptide glycan analysis. a Exemplary MS spectrum view of LC glycan peptide peaks shown and annotated in Fig. 2a. **b** Percentage of individual γHC glycan traits expressed on 2G9, 3F3 and D2 mIgG-BCRs and 2G9 sIgG. Bar graphs show mean, standard error and individual data points of 4 biological replicates. **c** Western blot analysis of FcG+ γHC after capturing of biotinylated mIgG (3F3) and EndoH (cleaving high mannose N-glycans) or PNGaseF (claving all N-glycans) treatment. Representative results of 2 biological replicates are shown. **d** Percentage of individual γHC glycan traits expressed on 2G9 mIgG-BCRs vs sIgG. Glycan traits are schematically depicted. Heat map shows mean of 4 biological replicates. Source data are provided as a Source Data file.

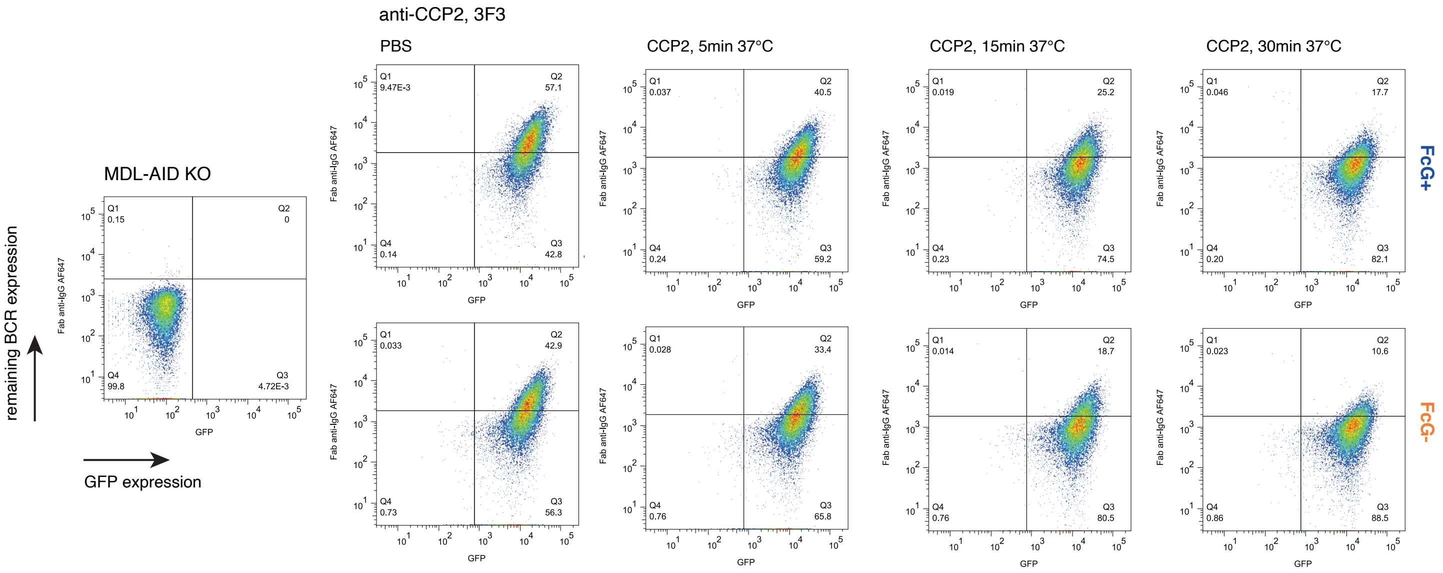

Supplementary Figure 3. IgG-BCR downmodulation after (antigenic) stimulation. Gating strategy to determine IgG-BCR downmodulation after activation (PBS or CCP2) in the presence and absence of FcG. Shown are the MDL-AID KO negative control and the anti-CCP2, 3F3 B-cell line.

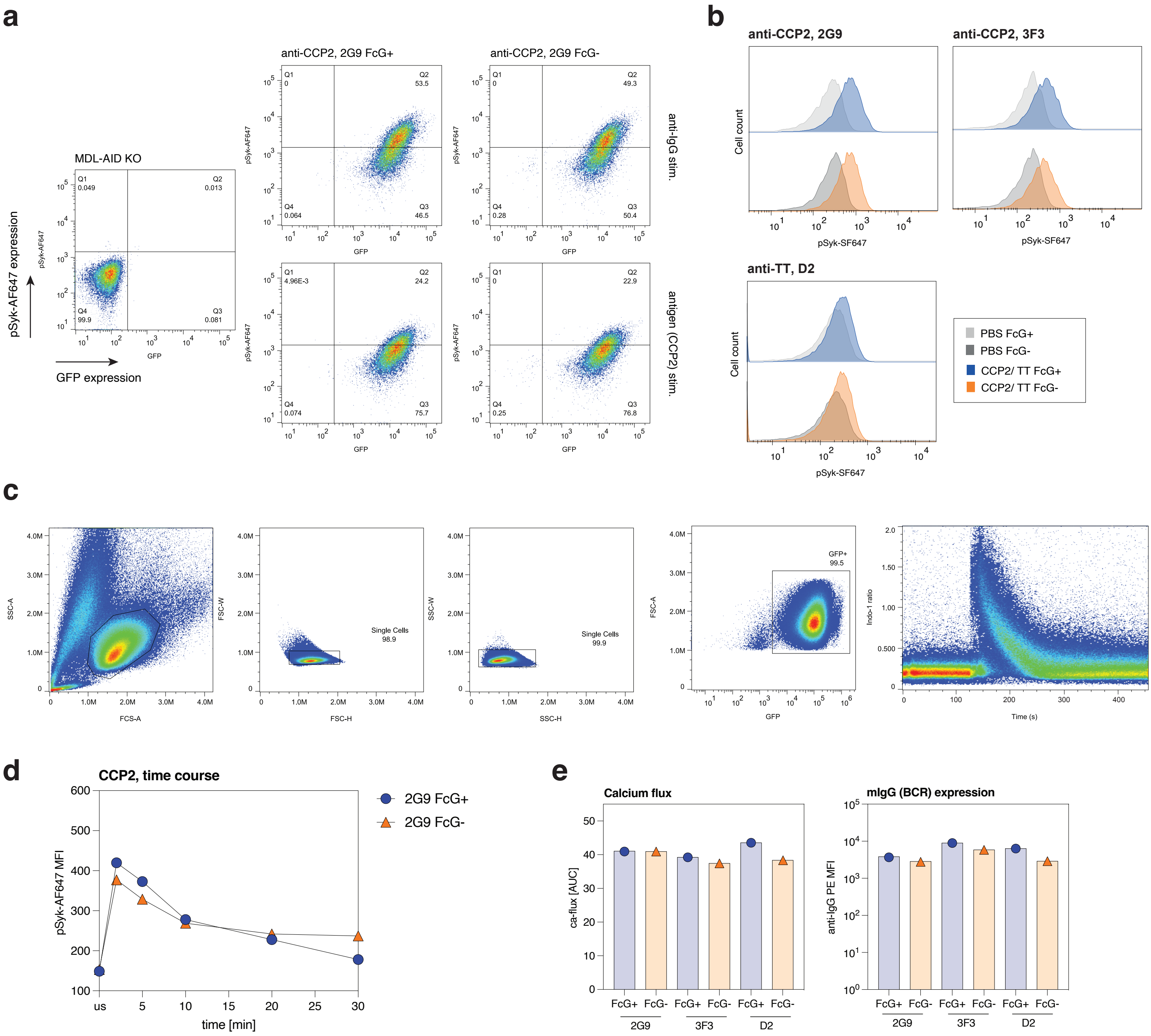

Supplementary Figure 4. Activation of mIgG-BCRs in the presence and absence of FcG. a Gating strategy to determine the pSyk expression after activation (anti-IgG or antigen) in the presence and absence of FcG. Shown are the MDL-AID KO negative control and the anti-CCP2, 2G9 B-cell line. **b** Flow histograms of pSyk expression of 2G9, 3F3 and D2 FcG+ and FcG- B cells after PBS treatment and antigenic stimulation. **c** Gating strategy to determine the calcium flux after activation (anti-IgG) in the presence and absence of FcG. Shown is the anti-CCP2, 2G9 FcG+ B-cell line. **d** pSyk MFI values of 2G9 FcG+ and FcG- B cells unstimulated (us) and after 5 – 30 min stimulation with 5 µg/ml CCP2. Representative results of 2 biological replicates are shown. **e** Calcium flux (AUC) and mIgG-BCR expression of FcG+ and FcG- B cell lines (2G9, 3F3 and D2). Measurements were performed within the same experiment. Source data are provided as a Source Data file.

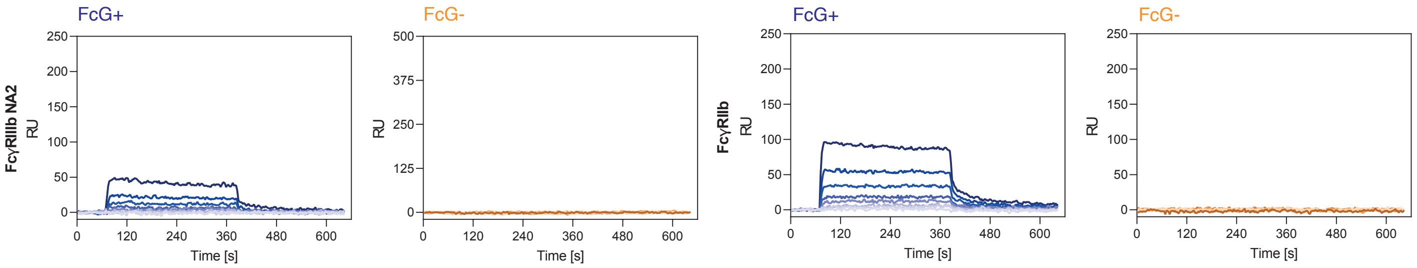

Supplementary Figure 5. Impact of IgG FcG on binding to FcɣRII/ III b. Representative SPR sensorgrams of 2G9 sIgG FcG+ and FcG- binding to human FcɣRII/ III b. Association and dissociation are represented as response units [RU] over time [s]. Source data are provided as a Source Data file.
