# Supplementary material for: N-linked Fc glycosylation is not required for IgG-B-cell receptor function in a GC-derived B-cell line
Source: Nat Commun. 2024 Jan 9;15:393. doi: 10.1038/s41467-023-44468-5 (PMC10776614; doi:10.1038/s41467-023-44468-5)
Supplement: Supplementary file 3 — Reporting Summary [file 41467_2023_44468_MOESM3_ESM.pdf]

nature portfolio

Corresponding author(s): Dr. Theresa Kissel; Prof. Dr. Rene E.M. Toes

Last updated by author(s): Nov 14, 2023

Reporting Summary

Nature Portfolio wishes to improve the reproducibility of the work that we publish. This form provides structure for consistency and transparency in reporting. For further information on Nature Portfolio policies, see our Editorial Policies and the Editorial Policy Checklist.

Statistics

For all statistical analyses, confirm that the following items are present in the figure legend, table legend, main text, or Methods section.

n/a	Confirmed	
☐	☒	The exact sample size (*n*) for each experimental group/condition, given as a discrete number and unit of measurement
☐	☒	A statement on whether measurements were taken from distinct samples or whether the same sample was measured repeatedly
☐	☒	The statistical test(s) used AND whether they are one- or two-sided<br>*Only common tests should be described solely by name; describe more complex techniques in the Methods section.*
☒	☐	A description of all covariates tested
☒	☐	A description of any assumptions or corrections, such as tests of normality and adjustment for multiple comparisons
☐	☒	A full description of the statistical parameters including central tendency (e.g. means) or other basic estimates (e.g. regression coefficient) AND variation (e.g. standard deviation) or associated estimates of uncertainty (e.g. confidence intervals)
☐	☒	For null hypothesis testing, the test statistic (e.g. *F*, *t*, *r*) with confidence intervals, effect sizes, degrees of freedom and *P* value noted<br>*Give P values as exact values whenever suitable.*
☒	☐	For Bayesian analysis, information on the choice of priors and Markov chain Monte Carlo settings
☒	☐	For hierarchical and complex designs, identification of the appropriate level for tests and full reporting of outcomes
☒	☐	Estimates of effect sizes (e.g. Cohen's *d*, Pearson's *r*), indicating how they were calculated

Our web collection on statistics for biologists contains articles on many of the points above.

Software and code

Policy information about availability of computer code

Data collection	LC-MS (Fc peptide glycan) data were collected using Compass 1.9 for OTOF version 4.0.15.3248 (Bruker Daltonik GmbH).
Data analysis	Flow cytometry data were analyzed using FlowJo_V10. LC-MS (Fc peptide glycan) data assessment and processing was performed using DataAnalysis (Bruker Daltonics) and LaCyTools v1.1.0 (https://github.com/Tarskin/LaCyTools). SPR data were analyzed using SPRINT 1.9.4.4 software (IBIS technologie) as well as Scrubber software version 2 (Biologic Software).

For manuscripts utilizing custom algorithms or software that are central to the research but not yet described in published literature, software must be made available to editors and reviewers. We strongly encourage code deposition in a community repository (e.g. GitHub). See the Nature Portfolio guidelines for submitting code & software for further information.

Data

Policy information about availability of data

All manuscripts must include a data availability statement. This statement should provide the following information, where applicable:

- Accession codes, unique identifiers, or web links for publicly available datasets
- A description of any restrictions on data availability
- For clinical datasets or third party data, please ensure that the statement adheres to our policy

We confirm that the data supporting the findings of this study are available within the article and/or can be provided upon request. The processed flow cytometry

data and LC-MS glycan data are provided in the Source Data file. The Ramos MDL-AID KO cells can be provided by Prof. Dr. Reth (Albert-Ludwigs-Universität Freiburg, Germany).

Research involving human participants, their data, or biological material

Policy information about studies with human participants or human data. See also policy information about sex, gender (identity/presentation), and sexual orientation and race, ethnicity and racism.

Reporting on sex and gender	n.a.
Reporting on race, ethnicity, or other socially relevant groupings	n.a.
Population characteristics	n.a.
Recruitment	n.a.
Ethics oversight	n.a.

Note that full information on the approval of the study protocol must also be provided in the manuscript.

Field-specific reporting

Please select the one below that is the best fit for your research. If you are not sure, read the appropriate sections before making your selection.

☒ Life sciences ☐ Behavioural & social sciences ☐ Ecological, evolutionary & environmental sciences

For a reference copy of the document with all sections, see nature.com/documents/nr-reporting-summary-flat.pdf

Life sciences study design

All studies must disclose on these points even when the disclosure is negative.

Sample size	No sample-size calculation was performed. All experiments are based on human B-cell line data and three different B-cell lines expressing mIgG BCRs in the presence or absence of Fc glycans were generated. In all experiments all three B-cell lines were assessed. In addition biological replicates of all three cell lines were performed for all experiments providing in total a sufficient sample size to identify functional differences between Fc glycosylated and non-glycosylated mIgG-BCRs. One B-cell line secreting IgG with or without Fc glycans was generated to support the known effect of Fc glycans on IgG effector functions. As this information is already known from literature one B-cell line was sufficient.
Data exclusions	No data were excluded from the analyses.
Replication	The reproducibility of the experimental findings was verified by using multiple human (Ramos) B-cell lines transduced with different IgG B-cell receptors in the presence or absence of Fc glycans. Reproducibility of all experiments using the generated Ramos cell lines were confirmed by biological or technical replications.
Randomization	Randomization was not applicable to this study. B-cell lines were specifically generated to either express or do not express Fc glycans.
Blinding	Blinding was not performed in this study. B-cell lines had to be assessed for the expression of the 'correct' Fc glycosylated or non-glycosylated mIgG or sIgG to exclude contaminations. Experiments were however replicated by different researchers.

Behavioural & social sciences study design

All studies must disclose on these points even when the disclosure is negative.

Study description	n.a.
Research sample	n.a.
Sampling strategy	n.a.
Data collection	n.a.
Timing	n.a.
Data exclusions	n.a.

Non-participation	n.a.
Randomization	n.a.

Ecological, evolutionary & environmental sciences study design

All studies must disclose on these points even when the disclosure is negative.

Study description	n.a.
Research sample	n.a.
Sampling strategy	n.a.
Data collection	n.a.
Timing and spatial scale	n.a.
Data exclusions	n.a.
Reproducibility	n.a.
Randomization	n.a.
Blinding	n.a.

Did the study involve field work? ☐ Yes ☐ No

Field work, collection and transport

Field conditions	n.a.
Location	n.a.
Access & import/export	n.a.
Disturbance	n.a.

Reporting for specific materials, systems and methods

We require information from authors about some types of materials, experimental systems and methods used in many studies. Here, indicate whether each material, system or method listed is relevant to your study. If you are not sure if a list item applies to your research, read the appropriate section before selecting a response.

Materials & experimental systems

n/a	Involved in the study	
☐	☒	Antibodies
☐	☒	Eukaryotic cell lines
☒	☐	Palaeontology and archaeology
☒	☐	Animals and other organisms
☒	☐	Clinical data
☒	☐	Dual use research of concern
☒	☐	Plants

Methods

n/a	Involved in the study	
☒	☐	ChIP-seq
☐	☒	Flow cytometry
☒	☐	MRI-based neuroimaging

Antibodies

Antibodies used	B-cell lines were sorted using AF647 NHS ester (Thermo Fisher; A2006) labelled Fab fragment goat anti-human IgG (Jackson ImmunoResearch; 109-007-003).<br>For flow cytometry experiments, B cells were stained with goat anti-human IgG-Fc phycoerythrin (PE) (eBioscienceTM; 12-4998-82; lot: 2481260), AF647 NHS ester (Thermo Fisher Scientific; A20006) labelled Fab goat anti-human IgG (Jackson ImmunoResearch;

	109-007-003) or mouse anti-human pSyk(Y319)-AF647 mAb (17A/P-ZAP70; BD; 557817; lot: 9165873). B cell stimulation was performed using goat F(ab')2 anti-human kappa (SouthernBiotech; 2062-01) or goat anti-human IgG F(ab')2 (Jackson ImmunoResearch; 109-006-097, lot: 164786). For Western blot analysis HC were detected using goat anti-human IgG (Invitrogen; 31410).<br>Enzyme-linked immunosorbent assays (ELISA) were performed using an HRP-conjugated rabbit-anti-human IgG secondary detection antibody (DAKO; P0214; lot: 20036015). To determine classical complement activation rabbit anti-C1q (DAKO; A0136), goat anti-C4 (QUIDEL; A305) or rabbit anti-C3c (DAKO; A0062) antibodies were used and binding was detected with HRP-labelled goat anti-rabbit (DAKO; P0448) or rabbit anti-goat (DAKO; P0449) detection antibodies. For SPR measurements biotinylated anti-His mIgG1 (GenScript; A00613) was used to load His-tagged hFcγRI onto the sensor.
Validation	The specificity of the antibodies used for the flow cytometry experiments was determined using IgG BCR negative control B cells (MDL-AID KO). Further validation of goat anti-human IgG (Jackson ImmunoResearch; 109-007-003) can be found on the suppliers website (including 5 citations). Further validation of goat anti-human IgG-Fc phycoerythrin (PE) (eBioscienceTM; 12-4998-82) can be found on the suppliers website (including 75 citations). Further validation of goat F(ab')2 anti-human kappa (SouthernBiotech; 2062-01) can be found on the suppliers website (including 14 citations). Further validation of goat anti-human IgG F(ab')2 (Jackson ImmunoResearch; 109-006-097) can be found on the suppliers website (including 19 citations). Further validation of mouse anti-human pSyk(Y319)-AF647 mAb (17A/P-ZAP70; BD; 557817) can be found on the suppliers website (including 3 citations).<br>The goat anti-human IgG (Invitrogen; 31410) antibody used for Western blot analyses was validated using non IgG containing control lanes. Further validation can be found on the suppliers website.<br>Complement activation ELISA antibodies were here validated based on the presence or absence of IgG antibodies and normal human serum (exogeneous complement). In addition the primary ELISA antibodies were validated in the previously performed study by 'Trouw, L. A. et al. Anti-cyclic citrullinated peptide antibodies from rheumatoid arthritis patients activate complement via both the classical and alternative pathways. Arthritis Rheum 60, 1923-1931 (2009). https://doi.org:10.1002/art.24622'. The goat anti-C4 was further validated in the study by 'Wright, A., Morrison, S. Effect of C2 Associated Carbohydrate structure of Ig Effector function: studies with chimeric mouse human IgG1 Antibodies in glycosylation mutants of chinese hamster ovary cells, J. Immunol 160:3393-3402, 1998'.<br>The biotinylated anti-His mIgG1 antibody was validated here by His-tagged hFcγRI and subsequent IgG deposition on the SPR sensor chip. Further validation can be found on the suppliers website (including 7 citations).

Eukaryotic cell lines

Policy information about cell lines and Sex and Gender in Research

Cell line source(s)	The recombinant monoclonal antibodies were produced in FreestyleTM 293-F cells (Gibco).<br>The human Ramos B-cell lines expressing the murine cationic amino-acid transporter 1 (slc7a1) under blasticidine resistance to be able to infect them with Moloney Murine Leukemia Virus (MMLV)-based retrovirus particles, was provided by Dr. Engels, University Göttingen. The MDL-AID (IGHM, IGHD, IGLC and activation-induced cytidin deaminase, AID) knock-out (KO) variant of the slc7a1 expressing Ramos cells was generated by Dr. He, University Freiburg. The 2G9, 3F3 and D2 Ramos B-cell lines were generated by retroviral transductions of the MDL-AID KO Ramos cells expressing scl7a1 using Phoenix-ECO (ATCC; CRL-3212) cells.
Authentication	Inserts for all the generated human Ramos B-cell lines (2G9, 3F3 and D2) were verified by Sanger sequencing. No surface IgG-BCR expression of MDL-AID KO cells was verified by flow cytometry.
Mycoplasma contamination	I hereby confirm that all cell lines were tested negative for mycoplasma contamination.
Commonly misidentified lines<br>(See ICLAC register)	No commonly misidentified cell lines were used in this study.

Palaeontology and Archaeology

Specimen provenance	n.a.
Specimen deposition	n.a.
Dating methods	n.a.

☐ Tick this box to confirm that the raw and calibrated dates are available in the paper or in Supplementary Information.

Ethics oversight	n.a.

Note that full information on the approval of the study protocol must also be provided in the manuscript.

Animals and other research organisms

Policy information about studies involving animals; ARRIVE guidelines recommended for reporting animal research, and Sex and Gender in Research

Laboratory animals	n.a.

Wild animals	n.a.
Reporting on sex	n.a.
Field-collected samples	n.a.
Ethics oversight	n.a.

Note that full information on the approval of the study protocol must also be provided in the manuscript.

Clinical data

Policy information about clinical studies

All manuscripts should comply with the ICMJE guidelines for publication of clinical research and a completed CONSORT checklist must be included with all submissions.

Clinical trial registration	n.a.
Study protocol	n.a.
Data collection	n.a.
Outcomes	n.a.

Dual use research of concern

Policy information about dual use research of concern

Hazards

Could the accidental, deliberate or reckless misuse of agents or technologies generated in the work, or the application of information presented in the manuscript, pose a threat to:

No	Yes	
☒	☐	Public health
☒	☐	National security
☒	☐	Crops and/or livestock
☒	☐	Ecosystems
☒	☐	Any other significant area

Experiments of concern

Does the work involve any of these experiments of concern:

No	Yes	
☒	☐	Demonstrate how to render a vaccine ineffective
☒	☐	Confer resistance to therapeutically useful antibiotics or antiviral agents
☒	☐	Enhance the virulence of a pathogen or render a nonpathogen virulent
☒	☐	Increase transmissibility of a pathogen
☒	☐	Alter the host range of a pathogen
☒	☐	Enable evasion of diagnostic/detection modalities
☒	☐	Enable the weaponization of a biological agent or toxin
☒	☐	Any other potentially harmful combination of experiments and agents

Plants

Seed stocks	n.a.
Novel plant genotypes	n.a.
Authentication	n.a.

ChIP-seq

Data deposition

☐ Confirm that both raw and final processed data have been deposited in a public database such as GEO.

☐ Confirm that you have deposited or provided access to graph files (e.g. BED files) for the called peaks.

Data access links<br>*May remain private before publication.*	n.a.
Files in database submission	n.a.
Genome browser session<br>(e.g. UCSC)	n.a.

Methodology

Replicates	n.a.
Sequencing depth	n.a.
Antibodies	n.a.
Peak calling parameters	n.a.
Data quality	n.a.
Software	n.a.

Flow Cytometry

Plots

Confirm that:

☒ The axis labels state the marker and fluorochrome used (e.g. CD4-FITC).

☒ The axis scales are clearly visible. Include numbers along axes only for bottom left plot of group (a 'group' is an analysis of identical markers).

☒ All plots are contour plots with outliers or pseudocolor plots.

☐ A numerical value for number of cells or percentage (with statistics) is provided.

Methodology

Sample preparation	To analyze membrane-bound IgG (BCR) expression of transduced Fc glycan positive and Fc glycan negative 2G9, 3F3 and D2 GFP+ B-cell lines and the non-transduced MDL-AID KO GFP- B-cell line, cells were stained with 0.5 µg/ml goat anti-human IgG-Fc phycoerythrin (PE) (eBioscienceTM; 12-4998-82) in staining solution (PBS/0.5%BSA/ 0.02% NaN3) on ice for 30 min. To determine antigen binding, B cells were stained with Allophycocyanin (APC)-labelled CCP2/CArgP2-strep. tetramers (0 to 5 µg/ml) or APC-TT on ice for 30 min. For anti-CCP2 B-cell lines binding to the CArgP2 negative-control antigen was subtracted.<br>To determine BCR downmodulation after stimulation, 0.2 million B cells were incubated for 30 min on ice followed by 15 min stimulation at 4°C either with control PBS, CCP2-/CArgP2-strep. tetramers (5 µg/ml), TT-strep.tetramers (1.5 µg/ml) or 1 µg/ ml goat F(ab')2 anti-human kappa (SouthernBiotech; 2062-01) in PBS/2% FCS. Stimulated B cells were incubated for 0, 5, 15 or 30 min at 37°C to allow BCR-antigen uptake. The surface remaining IgG-BCRs were stained with AF647 NHS (N-hydroxysuccinimide) ester (Thermo Fisher Scientific; A20006) labelled Fab goat anti-human IgG (Jackson ImmunoResearch; 109007003) diluted 1:2000 in staining solution.<br>For IgG-BCR activation, 0.3 million B cells were stimulated with 5 µg/ml CCP2-strep. tetramer, TT-strep. tetramer or goat anti-human IgG F(ab')2 (Jackson ImmunoResearch; 109-006-097) for 5 min at 37°C in stimulation medium (RPMI/100 U/ml P/S/ GlutaMAXTM/10 mM Hepes/1% FCS). Afterward, cells were fixed (BioLegend Fixation Buffer; 420801) and permeabilized (True-PhosTM Perm Buffer; 425401). After washing, the intracellular expression of phosphorylated Syk was determined with a mouse anti-human pSyk(Y319)-AF647 mAb (BD; 17A/P-ZAP70, BD) diluted 1:10 in staining solution.<br>For calcium flux measurements, 1 million B cells were stained in 200 µl calcium- indicator loading dye medium containing 2 µM Indo-1 AM (Abcam; ab142778) and 0.05% pluronic acid (Molecular Probes; P6866) in stimulation medium (RPMI1640/100 U/ml P/S/GlutaMAXTM/10 mM Hepes/2%F°S) for 35 min at 37°C in the dark. After washing, B cells were incubated with 500 µl stimulation medium plus 2 mM calcium on ice and in the dark until usage. 15 min before the flow analysis, B cells were prewarmed at 37°C to decrease baseline activation upon measurement. After ~1.5 min of baseline measurement, PBS or 20 µg/ml goat anti-human IgG F(ab')2 (final concentration) (Jackson ImmunoResearch; 109-006-097) was added and mixed adequately. The measurement continued for another 6 min until the signal reached baseline again. Calcium flux was measured as the ratio of calcium-bound Indo-1 to unbound Indo-1.
Instrument	Stained cells were analyzed on a BD LSR-II or a Cytek Aurora 5L flow cytometry instrument.

Software | Data were analyzed with FlowJo_V10.

Cell population abundance | Transduced Ramos B cells were sorted based on GFP and membrane-bound IgG expression to ensure an identical amount of Fc glycan positive and Fc glycan negative IgG BCRs on the cell surface. The post-sort abundance of membrane-bound IgG and GFP double positive Ramos B cells was >94%.

Gating strategy | Gating was based on the IgG BCR negative (MDL-AID KO) control B-cell line.

☒ Tick this box to confirm that a figure exemplifying the gating strategy is provided in the Supplementary Information.

Magnetic resonance imaging

Experimental design

Design type: n.a.

Design specifications: n.a.

Behavioral performance measures: n.a.

Acquisition

Imaging type(s): n.a.

Field strength: n.a.

Sequence & imaging parameters: n.a.

Area of acquisition: n.a.

Diffusion MRI ☐ Used ☐ Not used

Preprocessing

Preprocessing software: n.a.

Normalization: n.a.

Normalization template: n.a.

Noise and artifact removal: n.a.

Volume censoring: n.a.

Statistical modeling & inference

Model type and settings: n.a.

Effect(s) tested: n.a.

Specify type of analysis: ☐ Whole brain ☐ ROI-based ☐ Both

Statistic type for inference (See Eklund et al. 2016): n.a.

Correction: n.a.

Models & analysis

n/a	Involved in the study
☐	☐ Functional and/or effective connectivity
☐	☐ Graph analysis
☐	☐ Multivariate modeling or predictive analysis

Functional and/or effective connectivity	n.a.
Graph analysis	n.a.
Multivariate modeling and predictive analysis	n.a.
